# Temporal variations in root-associated fungal communities of *Potaninia mongolica*, an endangered relict shrub species in the semi-arid desert of Northwest China

**DOI:** 10.3389/fpls.2022.975369

**Published:** 2022-10-12

**Authors:** Yonglong Wang, Ying Xu, Pulak Maitra, Busayo Joshua Babalola, Yanling Zhao

**Affiliations:** ^1^ Faculty of Biological Science and Technology, Baotou Teacher’s College, Baotou, China; ^2^ Institute of Dendrology, Polish Academy of Sciences, Kórnik, Poland; ^3^ State Key Laboratory of Mycology, Institute of Microbiology, Chinese Academy of Sciences, Beijing, China

**Keywords:** root-associated fungi, *potaninia mongolica*, relict plant, temporal dynamics, community assembly

## Abstract

The semi-arid region of the Western Ordos plateau in Inner Mongolia, China, is home to a critically endangered shrub species, *Potaninia mongolica*, which originates from ancient Mediterranean regions. Root-associated microbiomes play important roles in plant nutrition, productivity, and resistance to environmental stress particularly in the harsh desert environment; however, the succession of root-associated fungi during the growth stages of *P. mongolica* is still unclear. This study aimed to examine root-associated fungal communities of this relict plant species across three seasons (spring, summer and autumn) using root sampling and Illumina Miseq sequencing of internal transcribed spacer 2 (ITS 2) region to target fungi. The analysis detected 698 fungal OTUs in association with *P. mongolica* roots, and the fungal richness increased significantly from spring to summer and autumn. Eurotiales, Hypocreales, Chaetothyriales, Pleosporales, Helotiales, Agaricales and Xylariales were the dominant fungal orders. Fungal community composition was significantly different between the three seasons, and the fungal taxa at various levels showed biased distribution and preferences. Stochastic processes predominantly drove community assembly of fungi in spring while deterministic processes acted more in the later seasons. The findings revealed the temporal dynamics of root-associated fungal communities of *P. mongolica*, which may enhance our understanding of biodiversity and changes along with seasonal alteration in the desert, and predict the response of fungal community to future global changes.

## Introduction

Fungi are essential components of plant-associated microbiota, and play vital roles in regulating plant healthy, resistance to environmental stress, plant community dynamics and therefore ecosystem functioning (Robinson et al., 2020; Trivedi et al., 2020). The rapid development of DNA sequencing techniques has heightened attention to plant root-associated fungi in recent years (Qian et al., 2019; Toju et al., 2019; Li et al., 2021; Sweeney et al., 2021; Wang et al., 2021; Miyamoto et al., 2022). Previous studies indicated that biotic factors like host plants and abiotic variables such as geographic distance, edaphic and climatic variables could shape soil or root associated fungal diversity and community turnover, suggesting the adaption of fungi to surrounding environmental conditions (Wehner et al., 2014; Hu Y. et al., 2019; Jiang et al., 2021; Jiao et al., 2021; Li et al., 2021; Miyamoto et al., 2022). More importantly, season alternations could influence host plant growth which may consequently affect root-associated fungal communities (Courty et al., 2008; Courty et al., 2010). While the dynamics of root-associated fungi in agricultural and grassland ecosystems have received more attention in recent years (Gao et al., 2019; Deveautour et al., 2020; Babalola et al., 2022), to our knowledge, the temporal variability of root-associated fungal community in arid desert remain largely unknown, although some studies explored specific fungal guild or group such as arbuscular mycorrhizal fungi (AMF) and endophytic fungi (Zhu et al., 2021; Zhao et al., 2022). Additionally, considering the crucial roles of root-associated fungi to ecosystem services and the continued expansion of dryland in many regions, it is therefore necessary to understand how fungal community structure in the dryland habitats and their changes with seasonal alteration.

Previous studies indicated that diversity, community and related biological parameters of root-associated fungi respond significantly to seasonal changes (Courty et al., 2010). For example, Zhao et al. (2022) showed that colonization and spore densities of AMF associated with *Gymnocarpos przewalskii* changed significantly across seasons. Endophytic fungal communities of *Kalidium schrenkianum* roots were also shown to be shaped by season (Zhu et al., 2021). The temporal dynamics of root-associated fungal community may be related to changes in carbohydrates release by host plants, and environmental conditions such as temperature, precipitation and soil properties could also directly affect fungal growth (Dumbrell et al., 2011; Voříšková et al., 2014; Solly et al., 2017; Barnes et al., 2018; Miyamoto et al., 2018). As we mentioned above, the studies involved in root-associated fungi in semi-arid or arid deserts have commonly only focused on specific fungal groups such as mycorrhizal and endophytic fungi (León-Sánchez et al., 2018; Hu D. D. et al., 2019; Li et al., 2020; Zhu et al., 2021; Zhao et al., 2022). Other fungal guilds associated with plant roots include saprotrophic and pathogenetic fungi which have played important roles in plant performance and ecosystem processes (Liu et al., 2022), but are rarely explored in studies of desert ecosystems. As fungi associated with roots can benefit plant growth (Almario et al., 2017; Trivedi et al., 2020), inquiring about the entire fungal community of roots could provide important insights in fungal roles in plant resistance in harsh environments and in facing global environmental changes including drought and heat stress.

Revealing the mechanisms underlying community assembly is a central goal in microbial ecology and attracted much attention in recent years (Stegen et al., 2012; Zhou and Ning, 2017; Chen et al., 2019; Hussain et al., 2021). A common framework to describe the assembly processes underlying microbial communities includes deterministic and stochastic processes (Vellend et al., 2014; Zhou and Ning, 2017). Commonly, deterministic processes include nonrandom and niche-based mechanisms such as abiotic filtering and interspecific interactions (e.g. competition). The influence of abiotic filtering (e.g. soil properties and climatic conditions) on the fungal community depended on variations in resource availability and the adaptive ability of different fungal taxonomy (Courty et al., 2008; Pierre-Emmanuel et al., 2016). Additionally, competition among fungi derived priority effects also played a central role in determining fungal community assembly (Koide et al., 2005; Kennedy et al., 2009). By contrast, stochastic processes emphasize the role of probabilistic dispersal and ecological drift (Hubbell, 2001; Vellend et al., 2014). In recent decades, an increasing number of studies indicated that deterministic and stochastic processes simultaneously drive fungal community assembly, but the relative importance of these two processes is dependent on scales and habitats type (Schröter et al., 2019; Hussain et al., 2021; Zhang et al., 2021; Zheng et al., 2021). For example, soil fungal communities in the island were mainly governed by deterministic processes regardless of island type (Zheng et al., 2021). Stochastic processes dominantly shaped soil fungal communities along an altitudinal gradient in Tibetan plateau (Hussain et al., 2021). Similarly, soil fungal communities in mangrove sediments along a 9000 km coastline were predicted by stochastic processes (Zhang et al., 2021). Root-associated fungal diversity and community structure in semi-arid desert ecosystems gradually received much attention, particularly the endophytic and symbiotic fungi as mentioned above, but the mechanisms underlying community assembly of root-associated fungi, and the changes along with seasonal alteration remained unclear now.

*Potaninia mongolica* Maxim, belonging monotypic genus *Potaninia* (Family Rosace) is an endemic shrub species in Inner Mongolia, but a relict of ancient Mediterranean flora from the Tertiary period, which is distributed in semi-arid desert in western Ordos desert, Northwest China (Li et al., 2003; Zhu et al., 2016). As a perennial shrub species, *P. mongolica* played an important role in the prevention of soil erosion and desertification in the arid region (Zhu et al., 2016). However, with the increase of human activities and continuous grazing in the recent decade, the ecological environments of habitat for this plant species have been severely damaged and the growth of the population is under serious threat (Wang, 2005; Zhu et al., 2016). As a rare and endangered species, *P. mongolica* has been included in the List of National Key Protected Wild Plants in China (http://www.forestry.gov.cn/main/5461/20210908/162515850572900.html). The ecology and biology of *P. mongolica* (Li et al., 2003; Zhu et al., 2016) has been examined, but the root-associated fungal diversity and community structure of this endangered relict plant remains unknown.

To address this gap in our knowledge, this study aimed to address three questions: (1) What is the root-associated fungal diversity and community composition of *P. mongolica* in the semi-arid desert ecosystem? (2) How about the temporal variations of root-associated fungal diversity and composition of *P. mongolica*? (3) What are the mechanisms underlying community assembly of the root-associated fungi of *P. mongolica*? To address these questions, we collected root samples across three consecutive seasons (spring, summer and autumn) from *P. mongolica* populations in Western Ordos desert of Inner Mongolia, China, and examined fungal communities using Illumina Miseq sequencing techniques on fungal internal transcribed spacer region 2 (ITS2 region). Revealing the endangered relict plant-associated fungal diversity, composition and assembly mechanisms, and their temporal variations could provide the basis for the reconstruction of plant-microbiome associations for plant protection and ecological restoration.

## Materials and methods

### Study site and sampling

The sampling site was located in the Western Ordos desert of Inner Mongolia, China (107°0′23″E, 40°12′30″N). The area has a typically arid continental climate with a mean annual temperature of 6.8 °C and mean annual precipitation of 276 mm, and about 60% of total precipitation is concentrated in June, July and August. The sampling site is characterized by desert sands, and the vegetation is dominated by xerophytic shrubs, of which *P. mongolica* is the dominant shrub species. The sampled shrub individuals were about twenty years. Sample collection work was conducted in April (Spring), July (Summer) and September (Autumn), spanning three seasons in the semi-arid desert. At each sampling timepoint, the fine roots were collected from eight plots. In each plot (50 × 50 m), five healthy plant individuals with no clear disease spot were selected (at least 20 m apart). The upper layer of soil was removed to clear the litter, and the fine roots were obtained by using shovel from a depth of about 30-40 cm. The five repeating subsample (individual) collected from each plot were merged into one sample, and thus a total of 24 samples were obtained in present study. The distance between each plot was at least 0.5 km to enable the sample independence. The samples were stored in an ice-box and transferred to the laboratory within 24 hours. In the laboratory, the fine roots were gently washed under running tap water to clear the soil debris and stored at -80°C until genomics extraction.

### Molecular analysis

About 25g of fine roots were used for DNA extraction for each sample in our study. The fine roots were ground using liquid N_2_ in a sterilized mortar, and total DNA were extracted using Plant DNA Extraction kit (Tiangen Biotech Beijing, China) according to the manufacturer’s instruction. The fungal internal transcribed spacer 2 (ITS2) region of rDNA was amplified by using primer set gITS7 (5’-GTGARTCATCGARTCTTTG-3’) and ITS4 (5’- TCCTCCGCTTATTGATATGC-3’) (Ihrmark et al., 2012). The PCR products were purified using Wizard SV Gel and PCR Clean-Up System (Promega, Madison, WI, USA). The purified DNA concentration was determined by a NanoDrop 2000 UV-vis spectrophotometer (Thermo Scientific, Wilmington, CA, USA), after that, the purified PCR products were pooled with equimolar amounts (100 ng) from each sample and adjusted to 10 ng μL^−1^. The amplicon sequencing work was conducted by Chengdu Institute of Biology, Chinese Academy of Sciences, China on the Illumina MiSeq PE250 platform with the paired end (2 × 250 bp) option.

### Bioinformatic analysis

The raw sequence data were processed using QIIME v.1.9.0 platform (Caporaso et al., 2010). The details on quality-control, ITS2 extraction and chimera check have been described in Wang Z. H. et al. (2019). The remaining high-quality non-chimeric ITS2 sequences were clustered into operational taxonomic units (OTUs) according to a 97% similarity cutoff using UPARSE pipeline (Edgar, 2013) after dereplication and discarding all singletons. The taxonomy of the representative sequence of each OTU was analyzed by searching against the entries in the unified system for the DNA-based fungal species linked to the classification (UNITE) database (Kõljalg et al., 2013) by using the basic local alignment search tool (BLAST) (Altschul et al., 1990). After this, the fungal identification was performed according to the criteria proposed by Tedersoo et al. (2014). Fungal OTUs were then assigned to different ecological categories based on genus-level identification according to FungalTraits (Põlme et al., 2020). To eliminate the effect of different sequencing depths among samples on the data analysis, the number of sequences per sample was normalized to the smallest sample size by using the rrarefy command in the vegan package of R v. 3.5.1. (R Development Core Team, 2019). Raw sequences have been deposited in the Sequence Read Archive of NCBI under BioProject PRJNA826555.

### Statistical analyses

The fungal OTUs accumulation curves from each season (eight samples in each season) were calculated using the specaccum command in the vegan package (Oksanen et al., 2013). The data of fungal OTUs richness, relative abundances of abundant fungal taxa (number of sequences more than 5%) satisfied the tests for the normality of distribution and homogeneity of variance before and after logarithm or root square transformation were analyzed by using analysis of variance (ANOVA) and the significant differences among seasons were further compared using Tukey’s honestly significant difference (HSD) test at *P* < 0.05 level. For the data that did not satisfy normality of distribution and homogeneity of variance after transformation, nonparametric Kruskal-Wallis test was adopted, followed by pairwise comparisons between seasons by using the posthoc.kruskal.dunn.test command with Bonferroni correction in the PMCMR package (Pohlert, 2014). Venn diagram was drawn to examine the exclusive and shared OTUs among seasons by using online tool Evenn (Chen et al., 2021; http://www.ehbio.com/test/venn/#/).

A Bray-Curtis dissimilarity matrix of fungal community composition was calculated based on the Hellinger-transformed community matrix using vegdist commands in the vegan package (Oksanen et al., 2013), and then nonmetric multidimensional scaling (NMDS) ordination was used to examine the fungal community using the metaMDS command in the vegan package (Oksanen et al., 2013). Permutational multivariate analysis of variance (PerMANOVA) was employed to assess significant temporal variations using the adonis command in the vegan package based on 999 permutations (Oksanen et al., 2013). Biomarkers of fungi with significantly different relative abundance from phylum to genus levels among seasons were investigated by using linear discriminant analysis (LDA) effect size (LEfSe) (Segata et al., 2011), and the threshold for logarithmic LDA score was set to 4.0 (Qian et al., 2021; Kushwaha et al., 2022). To evaluate the preference between season and fungi, the preference analysis was implied using the bipartite package (Toju et al., 2016). Additionally, to determine the relative contribution of deterministic and stochastic processes to fungal community assembly, the normalized stochasticity ratio (NST) was calculated based on the fungal OTU matrix and groups by using tNST command in the NST package. The index ranges from 0 to 100%, where 0% indicates no contribution of stochasticity and 100% suggests the community is completely driven by stochastic processes, and with 50% as the boundary point between more deterministic (<50%) and more stochastic processes (>50%) (Ning et al., 2019). Similar analysis including diversity comparisons, NMDS ordination, PerMANOVA as well as NST analyses were also conducted on saprotrophic and pathogenetic fungi.

## Results

### Fungal database summary and diversity

**Figure 1:**
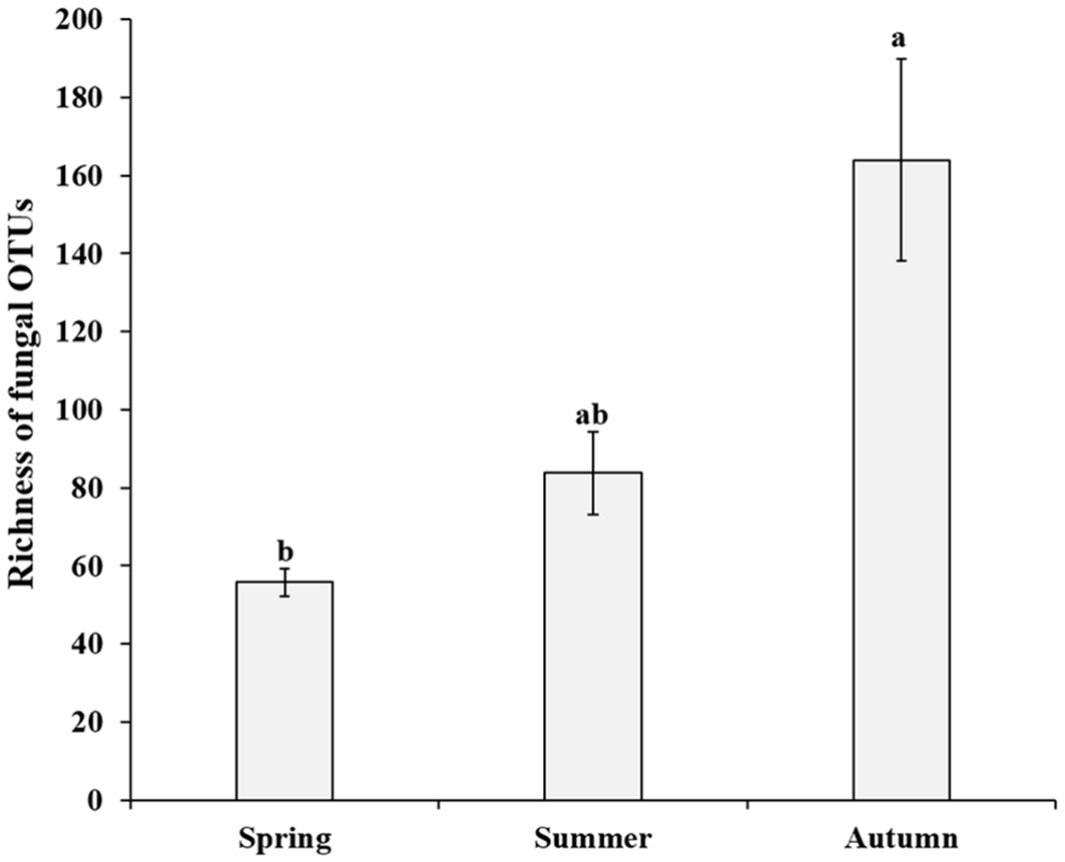
Richness of fungal operational taxonomic units (OTUs) of *P. mongolica* roots over three consecutive seasons. Bars without shared letters indicate significant differences in richness of fungal OTUs according to Tukey’s HSD at P < 0.05.

**Figure 2:**
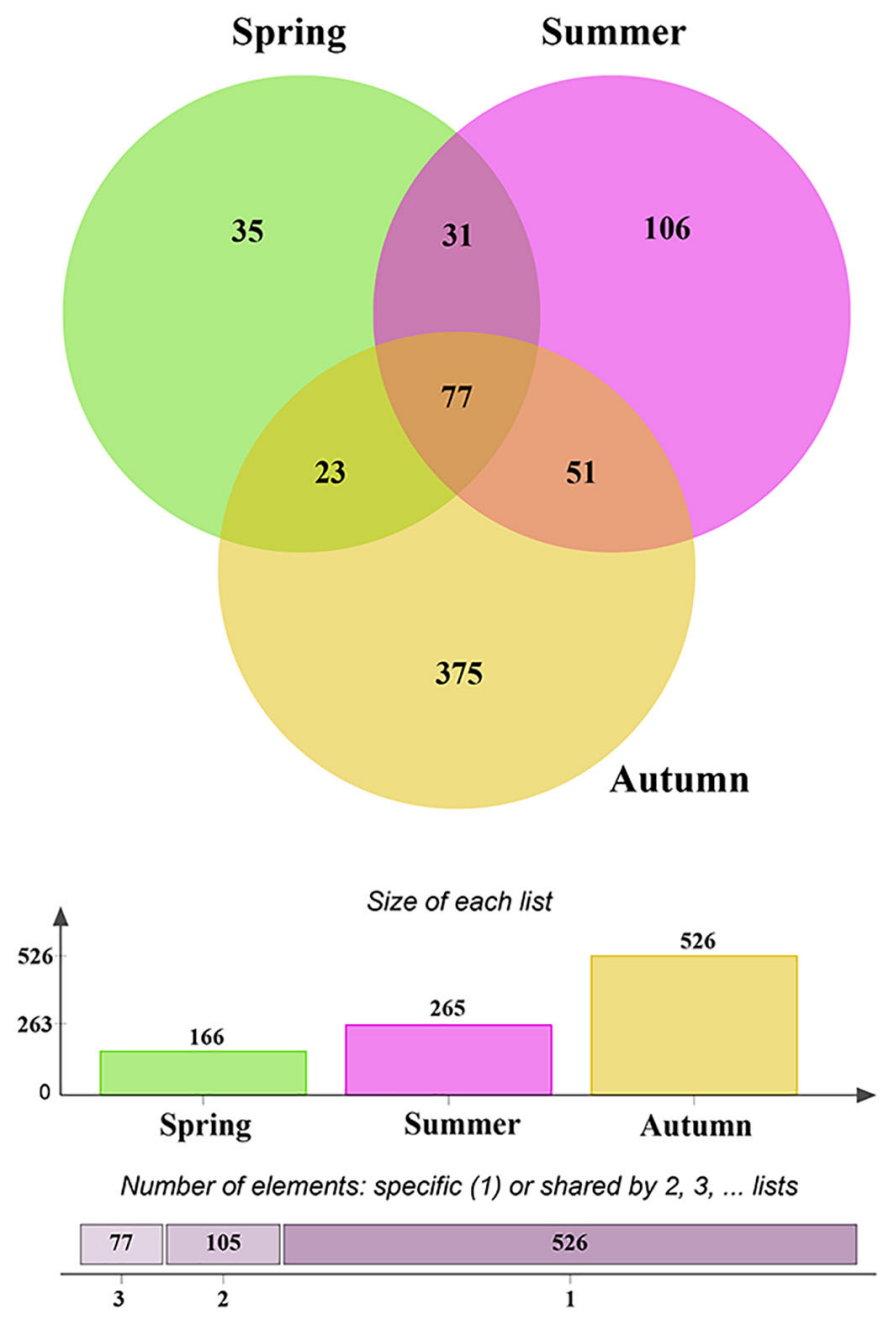
Venn diagram showing the shared and exclusive operational taxonomic units (OTUs) among seasons.

After quality control, a total of 1,468,500 non-chimeric ITS2 sequences were obtained (from 1,749,799 raw data), and the high-quality sequences were clustered into 1,363 OTUs, of which 1,075 were identified as fungal OTUs. After rarefaction, 698 fungal OTUs were reserved for further analysis. The OTUs accumulation curves for each season did not reach an asymptote, suggesting more plots collection could bring undiscovered fungal OTUs (**Supplementary Figure 1**). The richness of fungal OTUs ranged from 37 to 283 in all samples, and 55.8 ± 3.6 (Mean ± SE) in spring, 83.8 ± 10.6 in summer and 164 ± 25.9 in autumn, respectively. Kruskal-Wallis tests indicated that the fungal OTUs richness were significantly different across the three consecutive seasons (χ^2^ = 10.97, *P* = 0.004; **Figure 1**). Multiple comparisons analysis further showed that autumn samples harbored significantly higher fungal diversity than those in spring samples, but no significant difference between spring and summer samples (**Figure 1**). Similar temporal dynamics of fungal diversity were observed in saprotrophic and pathogenetic fungi (**Supplementary Figure 2**). Evenn showed that 77 fungal OTUs were shared by three seasons, with each season harboring 35, 106 and 375 unique OTUs, respectively, (**Figure 2**), which suggests that exclusive fungi were popular in certain season samples in present study.

### Fungal community composition and assembly

**Figure 3:**
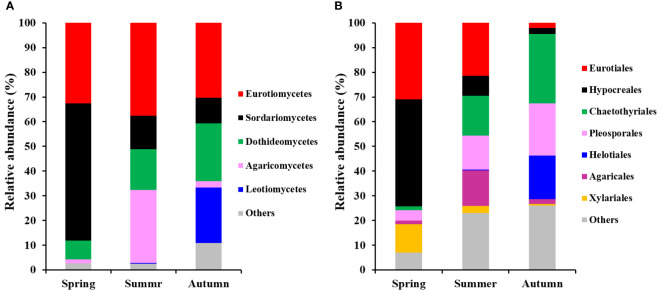
Relative abundances of the dominant fungal classes **(A)** and orders **(B)** found on roots of *P. mongolica* during 3 periods of the growth. Only the taxa consisting of > 5% of total sequences are shown.

Fungal classes Eurotiomycetes, Sordariomycetes, Dothideomycetes, Agaricomycetes and Leotiomycetes were dominant groups (each more than 5%), comprising 94.6% of total fungal reads in present study (**Figure 3A**). Meanwhile, Eurotiales, Hypocreales, Chaetothyriales, Pleosporales, Helotiales, Agaricales and Xylariales were the most dominant fungal orders, comprising 81.3% of total reads (**Figure 3B**).

**Figure 4:**
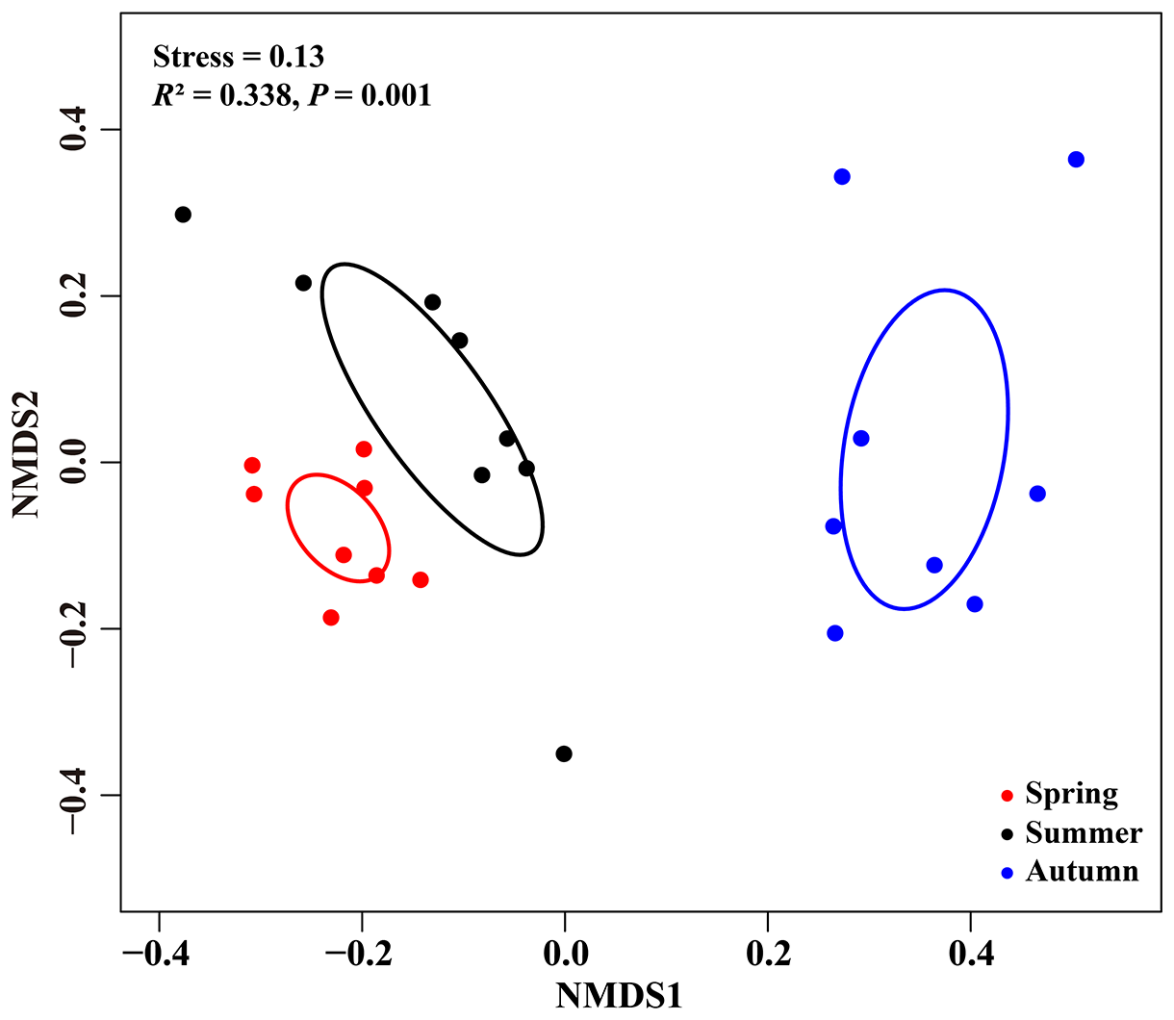
Nonmetric multidimensional scaling (NMDS) ordination of fungal community composition based on Bray-Curtis distance among seasons. Ellipses delimit 95% confidence intervals around centroids for each season.

**Figure 5:**
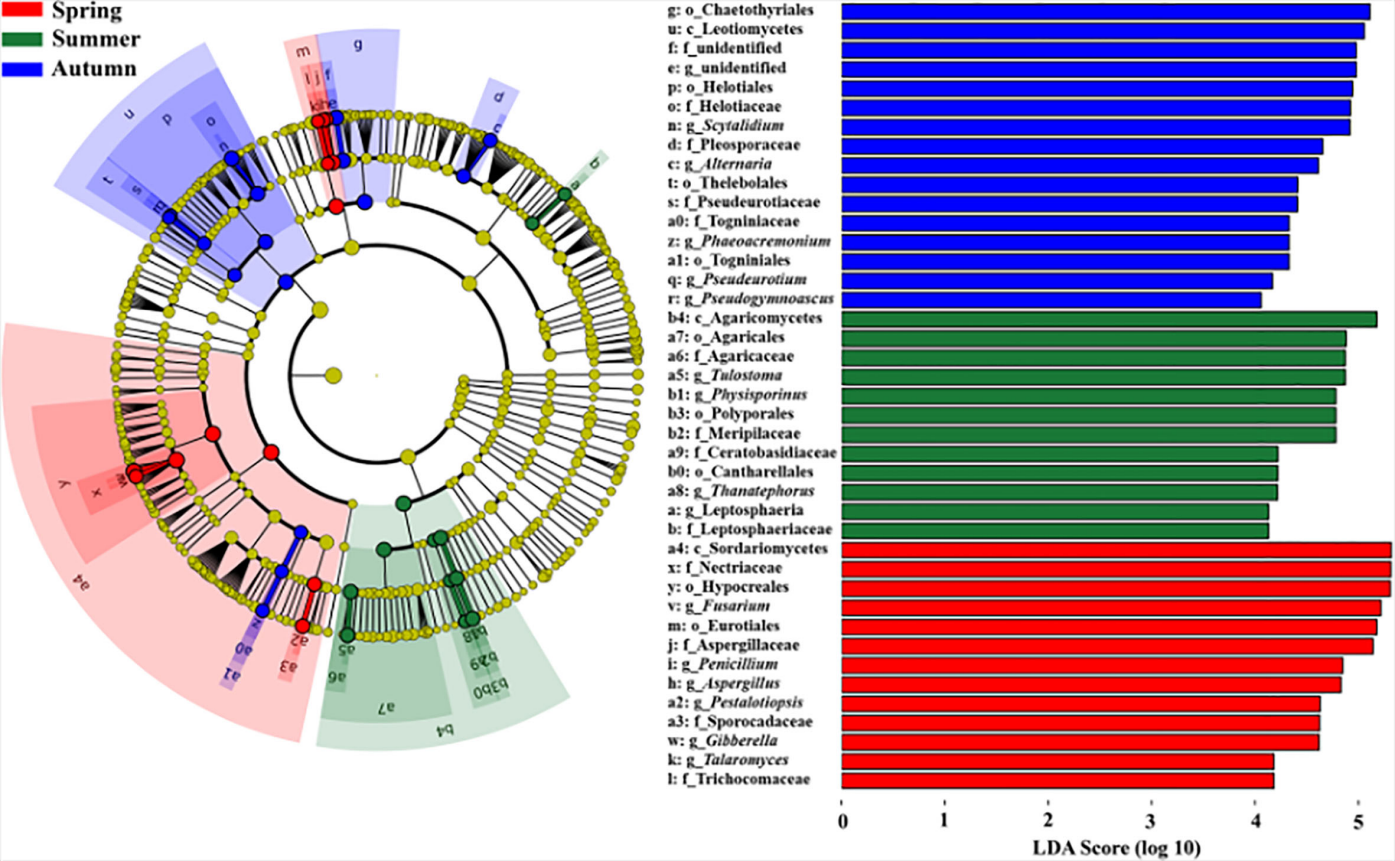
LDA effect size taxonomic cladograms and score histograms showing fungal indicator taxa of each season. The nodes from inside to outside indicate the taxonomic levels with Kingdom, Phylum (p_), Class (c_), Order (o_), Family (f_) and Genus (g_). The small yellow circles represent the fungal taxa with no significant differences, while small circles and sectors with other different colors represent the significantly enriched fungal taxa with a LDA value larger than 4.0 in different groups. The size of each small circle is roughly proportional to the relative abundance of that given taxa.

**Figure 6:**
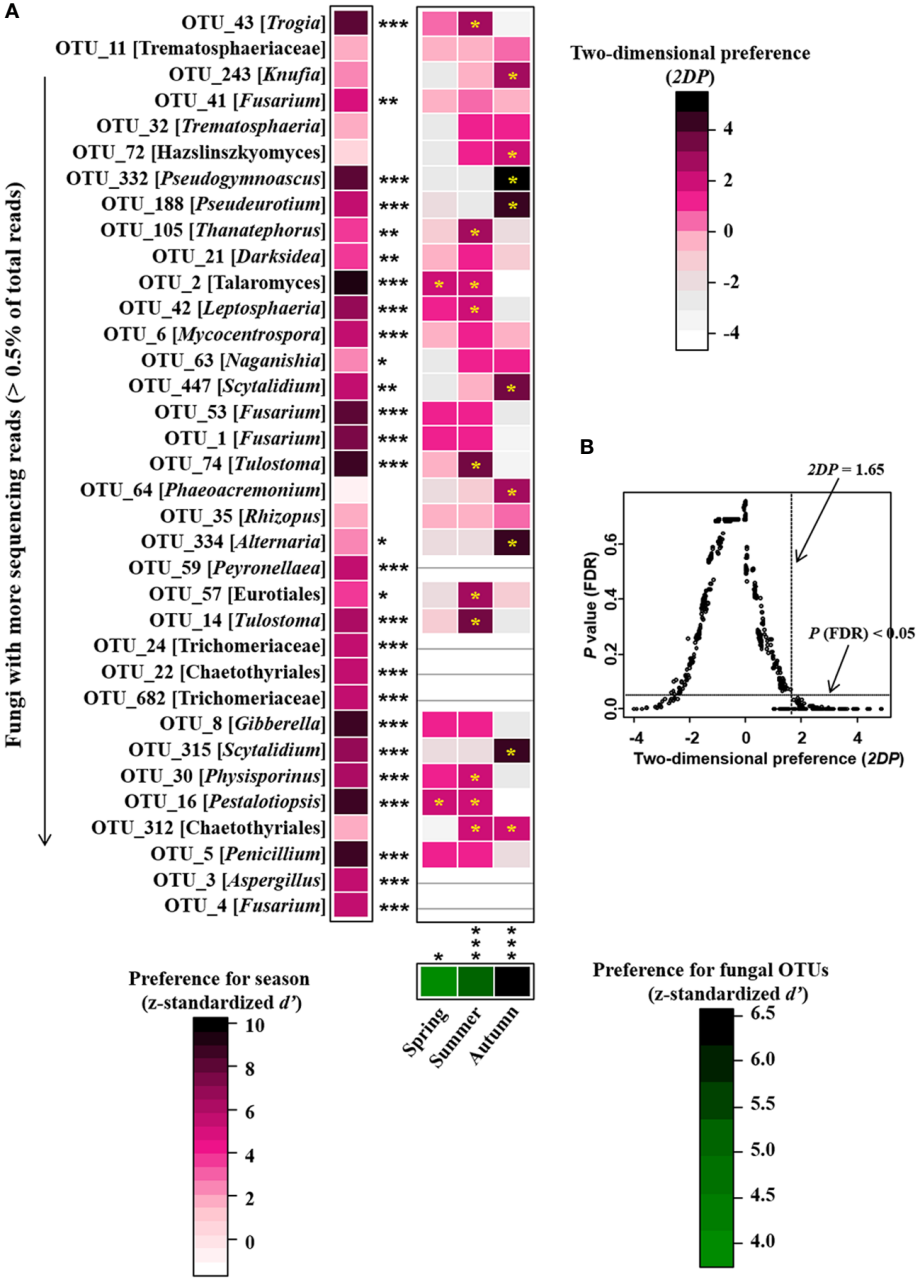
Preferences observed in season-fungus associations on roots of *P. mongolica*. **(A)** standardized *d’* estimates of preferences for fungal operational taxonomic units (OTUs) for indicated season (columns). Likewise, the standardized *d’* estimate of preferences for season is indicated for each of the observed fungal OTUs (row). A cell in the matrix indicates a two-dimensional preference (*2DP*) estimate, indicating the extent an association of a focal season-fungus pair was observed more/less frequently than expected by chance. The cell with asterisk inside represents significant preferences in season-fungus pair. Because multiple species/OTUs were tested, the *P* values are shown as false discovery rates (FDRs) in the season-fungus preference analysis. **(B)** relationship between *2DP* and FDR-adjusted *P* values, *2DP* values larger than 1.65 represented strong preferences. Significance: *P < 0.05, **P < 0.01, ***P < 0.001.

**Figure 7:**
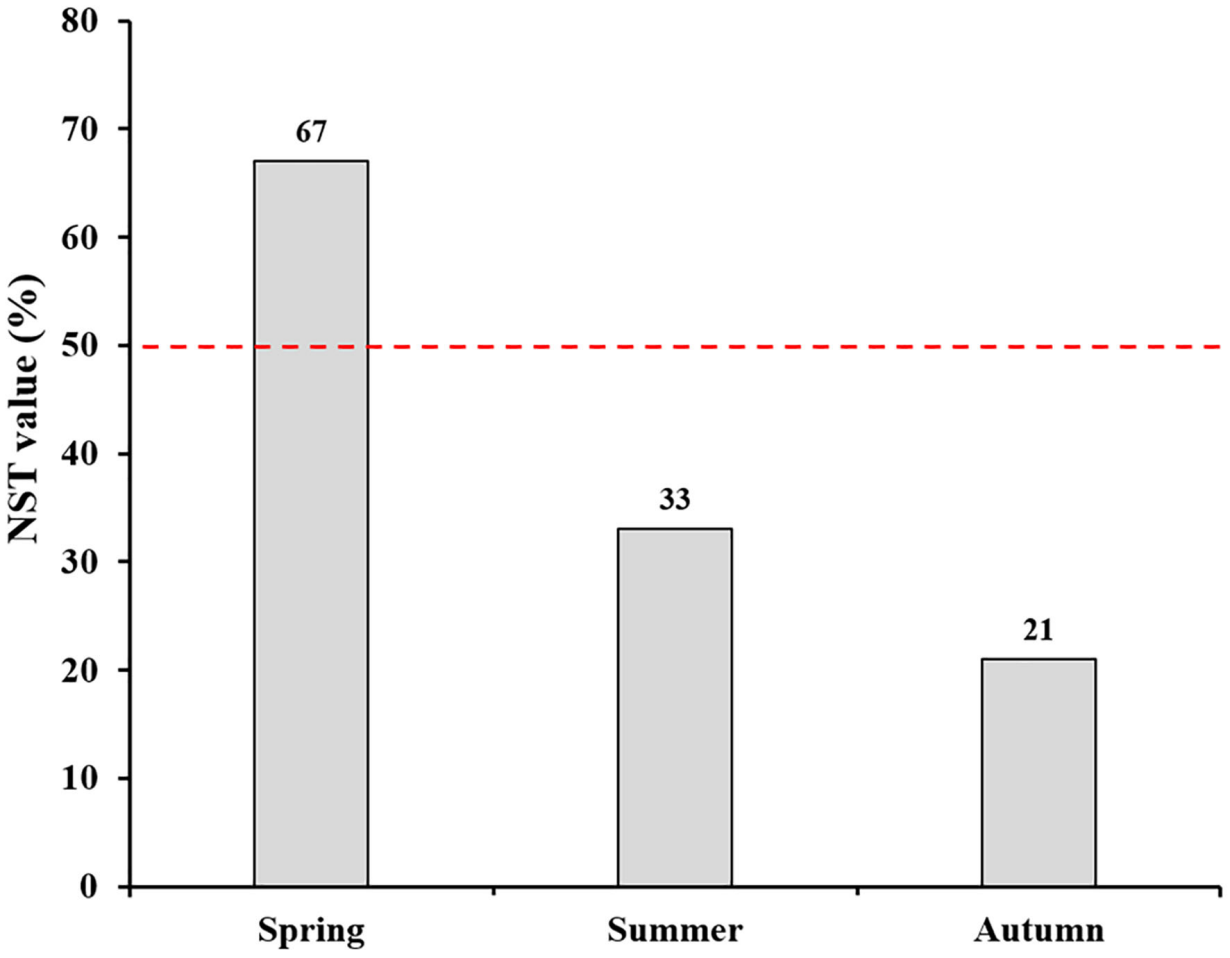
Normalized stochasticity ratio (NST) showing community assembly pattern of root-associated fungi of *P. mongolica* across three consecutive seasons.

NMDS ordinations showed that the fungal communities were clearly different across the three consecutive seasons (PerMANOVA: *R* ^2^ = 0.338, *P* = 0.001; **Figure 4**). Meanwhile, the community compositions of saprotrophic and pathogenetic fungi showed similar trends of temporal dynamics (**Supplementary Figure 3**). Three fungal classes (Sordariomycetes, Agaricomycetes and Leotiomycetes; **Supplementary Figure 4**) and five fungal orders (Eurotiales, Hypocreales, Chaetothyriales, Helotiales and Agaricales; **Supplementary Figure 5**) were distributed differently across three consecutive seasons; for example, the relative abundance of Sordariomycetes was significantly higher in spring than in seasons of summer and autumn (*F* = 11.07, *P* < 0.001; **Supplementary Figure 4A**) while Eurotiales was significantly lower in relative abundance in autumn than that in spring and summer (*F* = 10.04, *P* < 0.001; **Supplementary Figure 5A**). LefSe analysis indicated that 41 fungal taxa including three classes, nine orders, 13 families and 16 genera exhibiting significant taxonomic differences of relative abundance across seasons (**Figure 5**; **Supplementary Table 1**). For example, orders Eurotiales and Hypocreales, families Trichocomaceae, Sporocadaceae, Nectriaceae and Aspergillaceae, and genera *Talaromyces*, *Pestalotiopsis*, *Penicillium*, *Gibberella*, *Fusarium* and *Aspergillus* were the main discriminant taxon in spring samples (**Supplementary Table 2**). Preference analysis on season-fungi pairs showed that all season harbored certain fungal OTUs, 28 of 35 abundant OTUs (80.0%) existed in certain seasons, and 21 pairs of fungi and season (20.0%) showed significant partner existence (**Figure 6**). As for the certain fungal guilds, saprotrophic and pathogenic fungi were the dominant fungal guilds in the present study (**Supplementary Figure 6A**). The relative abundance of fungi identified as pathogenic was significantly different across seasons, with spring significantly higher than other seasons (*F* = 8.19, *P* = 0.002; **Supplementary Figure 6C**), but no significant differences was observed in saprotrophs across seasons. The average NST values of spring was 67%, followed by 33% and 21% in summer and autumn, which indicated that community assembly of fungi in spring were mainly controlled by stochastic process, while more driven by deterministic process in seasons of summer and autumn (**Figure 7**). As for the certain fungal guilds, that is, saprotrophic and pathogenetic fungi, the community assembly of which were mainly controlled by stochastics processes except saprotrophic fungi in the season autumn (**Supplementary Figure 7**).

## Discussion

Revealing the temporal variations of plant-associated fungi, exactly root-associated fungi in the current study, are important to investigate the roles of fungi in ecosystem functioning, as well as fungal responses to global climate changes (Vargas-Gastélum et al., 2015; Hu et al., 2021). *P. mongolica*, an endangered relict shrub plant received less attention in terms of fungal diversity and community assembly. Here we examined root-associated fungal diversity, communities, and assembly mechanisms across three consecutive seasons of this relict plant. A total of 698 fungal OTUs were detected in the current study. We speculated that the fungal diversity level of *P. mongolica* in this semi-arid desert was relatively lower than that detected in forest, grassland, wetland and agricultural ecosystems (Hu Y. et al., 2019; Wang Y. L. et al., 2019; Hussain et al., 2021; Jiao et al., 2021), suggesting the dry and low nutrients conditions in the semi-arid desert could not support a rich diversity of fungi. Indeed, a study conducted on diverse ecosystems in Hexi Corridor in the Gansu province in northwest China indicated that the fungal diversity significantly declined with the increasing of aridity (Jiao et al., 2021). In the present study, the fungal diversity increased significantly with season alteration from spring to summer and autumn, and reached its peak in the autumn. This trend of changes in fungal diversity here was partly consistent with Zhu et al. (2021), in which fungal diversity was highest in summer and autumn while relatively lower in spring and winter. The explanation may be that the growth and development of fungi were partly inhibited due to the harsh environments in terms of water and nutrient available from the soil and host plants in spring because the precipitation was very limited (only 9.5 mm) and the plant is in the sprouting stage. However, the plant could input more carbohydrates (e.g. exudates) into root and rhizosphere soil in summer due to relatively higher photosynthesis rate compared to in spring, and more litters were dedicated in autumn, these compounds are available for fungal growth and reproduction. Moreover, the temperature and precipitation are relatively higher in the late two seasons than in spring (see **Supplementary Table 3**), which is relatively proper for fungal growth, and thus more diverse fungi were supported (Voříšková et al., 2014; He et al., 2017).

Eurotiomycetes, Sordariomycetes, Dothideomycetes, Agaricomycetes and Leotiomycetes were the most dominant fungal classes while Eurotiales, Hypocreales, Chaetothyriales, Pleosporales, Helotiales, Agaricales and Xylariales were the dominant fungal orders in our study, which was similar with findings in previous studies those conducted in similar desert environments (Vargas-Gastélum et al., 2015; Zhang T. et al., 2016; Zuo et al., 2021). The fungal community structures were significantly different across three consecutive seasons, indicating strong temporal dynamics of fungal communities in present study, which was consistent with previous studies (Voříšková et al., 2014; Vargas-Gastélum et al., 2015; He et al., 2017). This may be ascribed to the changes of different fungal taxon across three seasons. Indeed, temporal dynamics of dominant fungal taxon have been observed in the current study, in detail, the relative abundances of three fungal classes (Sordariomycetes, Agaricomycetes and Leotiomycetes) and five fungal orders (Eurotiales, Hypocreales, Chaetothyriales, Helotiales and Agaricales) were significantly different across three consecutive seasons. The variations in abundance of fungal taxon across seasons has been further evidenced by the LefSe analysis, which indicated that 41 fungal taxa from class to genus level, in other world, three classes, nine orders, 13 families and 16 genera exhibited significant taxonomic differences across season samples. Additionally, based on the preference analysis, fungi also showed unbalanced distribution across seasons at the OTUs level. In our study, 28 of 35 abundant OTUs (80.0%) existed in specific season, and 21 pairs of fungi and seasons (20.0%) showed significant partner associations, this maybe also contributed to the dynamics of fungal community structure across seasons. Concerning the specific fungal functional guilds, the relative abundances also fluctuated across seasons. All in all, the differences of fungal taxon at various taxonomic levels along with season alteration contributed to the significant difference in fungal community structure across seasons. The changes of fungal taxon at various levels may be related to the variations in climatic conditions (e.g. precipitation and temperature), soil properties, and also plant primary productivity and released substrates, which have been reported in previous studies (Belnap et al., 2005; Zak, 2005; Vargas-Gastélum et al., 2015; Barnes et al., 2018; Shigyo et al., 2019). Fundamentally, the different distributions of fungal taxon across seasons could be caused by the difference in the suitable ability of fungi to environmental changes such as climatic and soil conditions and also plant growth stages, thus resulting in the selection pressure implemented by the host and environments, which has been observed and proved in previous studies (Solly et al., 2017; Miyamoto et al., 2018; Jiao et al., 2021; Zhao et al., 2022). Accordingly, exclusive fungal OTUs have been observed in our study, in detail, 516 OTUs (73.9%) only existed in one season and only 77 fungal OTUs (11.0%) were shared by the three seasons, and thus we speculated that most fungal OTUs occupied specific niches across season alteration.

Our analysis indicated that stochastic processes dominantly drove community assembly of root-associated fungi of *P. mongolica* in spring, while deterministic processes played more important roles than stochastic processes in the seasons of summer and autumn, which was consistent with studies conducted on annual crops. For example, Gao et al. (2020) suggested that stochastic process dominantly drove the community assembly of fungi in the roots and leaves of sorghum in the early growth stages of plants. Similarly, maize-associated fungal communities were more strongly driven by stochastic processes during early growth stages while deterministic processes predominately acted at the late stage. Thus, our study combined with studies mentioned above suggested that stochastic processes dominantly controlled fungal community assembly at the early growth stage of plant while deterministic acted more at the later stages, and the ecological processes controlling fungal community assembly could change along with seasonal alteration or plant growth stages irrespective of whether the plants are annual or perennial. In our study, root-associated fungal diversity was relatively lower in spring samples than those in summer and autumn, implying the dominance of ecological processes is related to fungal diversity level. We suspected that the fungal diversity level may mirror the dominance of deterministic and stochastic processes in driving community assembly, that is, if diversity level increases, the ecological processes may shift from stochastic to deterministic. Indeed, a strong negative correlation between fungal OTUs richness and the stochasticity ratio was observed by Jiao et al. (2021), indicating that fungal communities with high fungal diversity were less affected by stochastic processes than those with low diverse fungi. When it comes to specific fungal guilds in our study (i.e. saprotrophic and pathogenetic fungi), their mechanisms underlying community assembly were not consistent with the total fungi accurately, which suggests that different assembly rules when considering various fungal guilds, but the reasons for this need further investigation in the future study.

We should acknowledge that limited efforts in our field work as the samples were collected in one year, and therefore no replicates for each season in our study, which hampered our efforts to investigate the specific effect of seasons on root-associated fungal communities. Moreover, fungal communities commonly showed spatial structure at scales ranging from local to global scales, indicating the effect of geographic distance on community assembly. In our study, geographic information of each plot was not recorded, thus we cannot explore the variations of root-associated fungal communities among sites in each season. Thus, in our future study, field works should be performed lasts for three years, that is, three replicates for each season, and detailed geographic information of each plot in each season should be recorded, to enable us to explore the influence of season, sites, and their interactions on root-associated fungal communities. Additionally, the number of AM fungal OTUs and their sequence abundance were limited in our study (15 OTUs and 154 sequences), although this shrub species belongs to the AM host plant (Soudzilovskaia et al., 2020). We speculate that this plant species may be poorly colonized by AM fungi in this desert ecosystem. Indeed, some previous studies indicated that the AM fungal diversity, spore density, and root colonization rate declined with the increasing of aridity along a natural precipitation gradient (Yang et al., 2011; Zhang J. et al., 2016). The fine root samples collected in our fieldwork were limited and entirely used for molecular analysis, and thus in our future study, more root samples should be collected for morphological analysis.

## Conclusions

In summary, 698 fungal OTUs were identified from *P. mongolica* roots in this desert, and the fungal richness significantly increased along a temporal sequence extending from spring to summer and autumn. Eurotiales, Hypocreales, Chaetothyriales, Pleosporales, Helotiales, Agaricales and Xylariales were the dominant fungal orders. Fungal communities were significantly different across three seasons, indicating significantly temporal dynamics. Community assembly of fungi also changed along with season alteration as the stochastic processes predominantly drove community assembly of fungi in spring while deterministic processes acted more in the later seasons. Our study shed light on the fungal diversity and underlying community assembly processes of fungi associated with endangered relict plant in the semi-arid desert. This effort will ultimately help to improve our understanding on the response of plant-associated fungi to future climate changes in the desert ecosystem.

## Data availability statement

The datasets presented in this study can be found in online repositories. The names of the repository/repositories and accession number(s) can be found below: https://www.ncbi.nlm.nih.gov/, PRJNA826555.

## Author contributions

YW and YZ conceived and design the study. YX and YZ conducted the field work and performed the lab experiment. YW analyzed and wrote the first manuscript. YW, PM and BB reviewed and edit the manuscript. All authors contributed to the article and approved the submitted version.

## Funding

This work was funded by the Scientific Research Project of Colleges and Universities in Inner Mongolia Autonomous Region (No. NJZY21029), the Inner Mongolia Natural Science Foundation (No. 2021BS03027), the Baotou Teacher’s College High-level Research Achievement Cultivation Project (No. BSYKJ2021-ZQ01), and the High-level Talents Introduced Scientific Research Startup Fund Project of Baotou Teacher’s College (No. BTTCRCQD2020-001).

## Acknowledgments

We are grateful to Prof. Erica B. Young from the Department of Biological Sciences, University of Wisconsin-Milwaukee, United States for her important suggestions on our manuscript and English improvement.

## Conflict of interest

The authors declare that the research was conducted in the absence of any commercial or financial relationships that could be construed as a potential conflict of interest.

## Publisher’s note

All claims expressed in this article are solely those of the authors and do not necessarily represent those of their affiliated organizations, or those of the publisher, the editors and the reviewers. Any product that may be evaluated in this article, or claim that may be made by its manufacturer, is not guaranteed or endorsed by the publisher.
