# Supplementary material for: Temporal variations in root-associated fungal communities of Potaninia mongolica, an endangered relict shrub species in the semi-arid desert of Northwest China
Source: Front Plant Sci. 2022 Oct 12;13:975369. doi: 10.3389/fpls.2022.975369 (PMC9597089; doi:10.3389/fpls.2022.975369)
Supplement: Supplementary file 1 [file DataSheet_1.docx]

**Supplementary Information for**

**Temporal variations in root-associated fungal communities of *Potaninia mongolica*, an endangered relict shrub species in the semi-arid desert of Northwest China**

**Yonglong Wang^1^, Ying Xu^1^, Pulak Maitra^2^, Busayo Joshua Babalola^2^, Yanling Zhao^1^***

^1^Faculty of Biological Science and Technology, Baotou Teacher's College, Baotou, Inner Mongolia, China

^2^Institute of Dendrology, Polish Academy of Sciences, Kórnik, Poland

^3^State Key Laboratory of Mycology, Institute of Microbiology, Chinese Academy of Sciences, Beijing, China

**Supplementary Tables 1-3**

**Supplementary Figures 1-7**

**Supplementary Tables**

| **Supplementary Table 1.** The number of fungal taxa enriched in each season. | | | |
| --- | --- | --- | --- |
| Taxon | Spring | Summer | Autumn |
| Phylum | 0 | 0 | 0 |
| Class | 1 | 1 | 1 |
| Order | 2 | 3 | 4 |
| Family | 4 | 4 | 5 |
| Genus | 6 | 4 | 6 |

**Supplementary Table 2.** Biomarkers from phylum to genus levels in different seasons detected by LefSe analysis in present study.

See excel file.

| **Supplementary Table 3.** Climatic parameters of three growth season in the arid desert in present study. | | |
| --- | --- | --- |
| Season\Parameter | Temperature (◦C) | Precipitation (mm) |
| Spring | 8.8 | 9.5 |
| Summer | 22.5 | 65.1 |
| Autumn | 15.1 | 36.2 |

**Supplementary Figures**


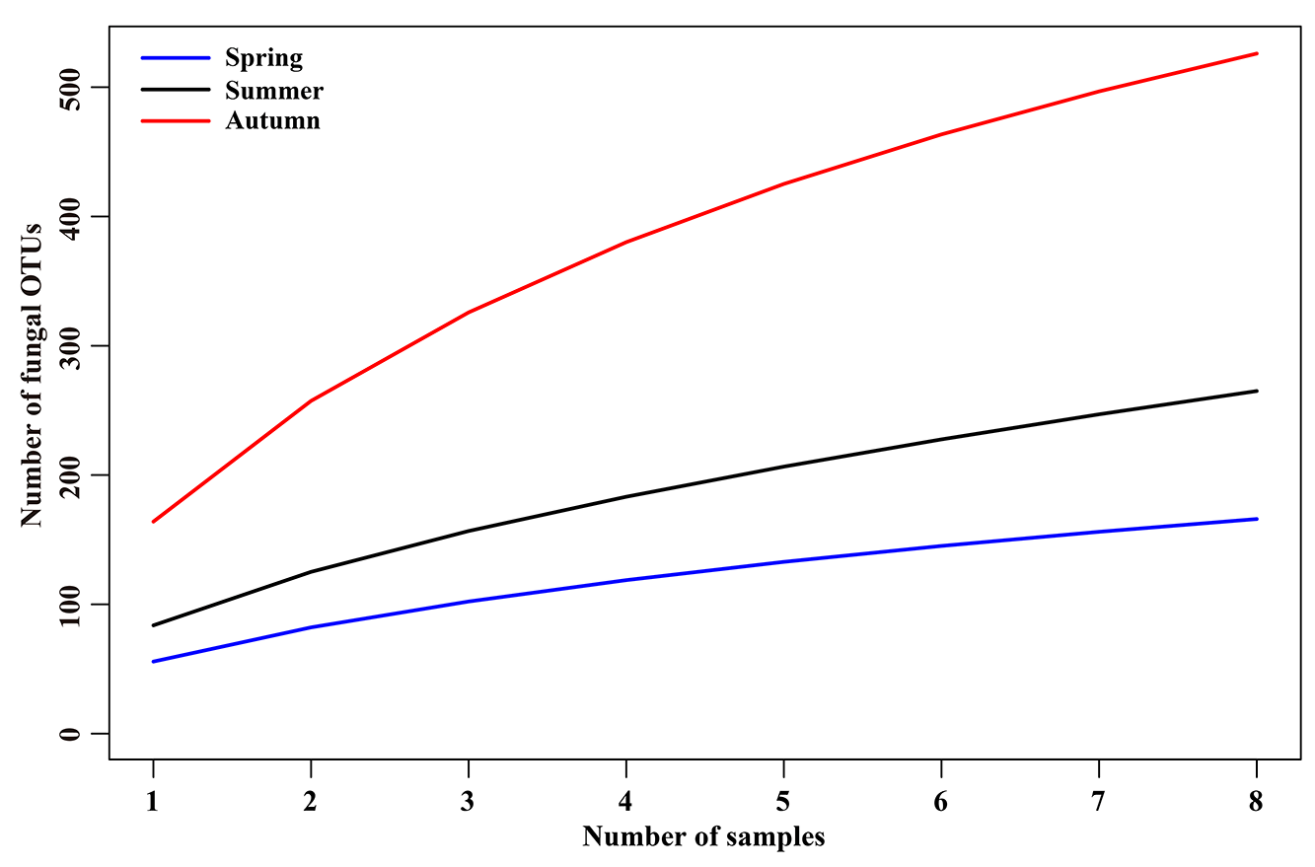


**Supplementary Figure 1.** Species accumulation curves of fungal operational taxonomic units (OTUs) in three seasons.


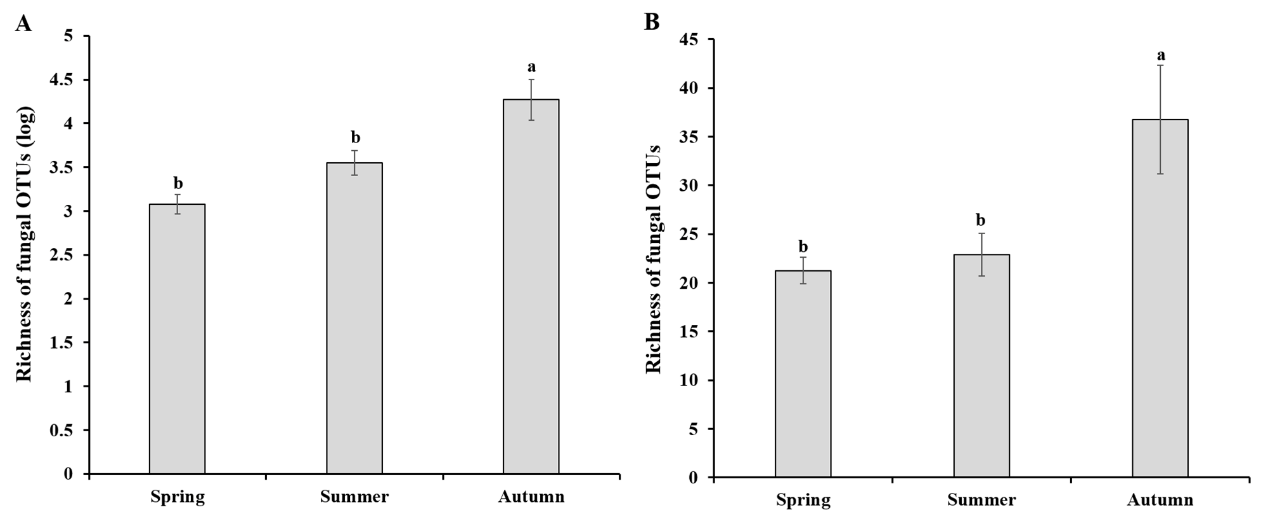


**Supplementary Figure 2.** Richness of operational taxonomic units (OTUs) of Saprotrophic fungi (A) and Pathogenetic fungi (B) over three growth seasons.. Bars without shared letters indicate significant differences in richness of fungal OTUs according to Tukey’s HSD at P < 0.05.


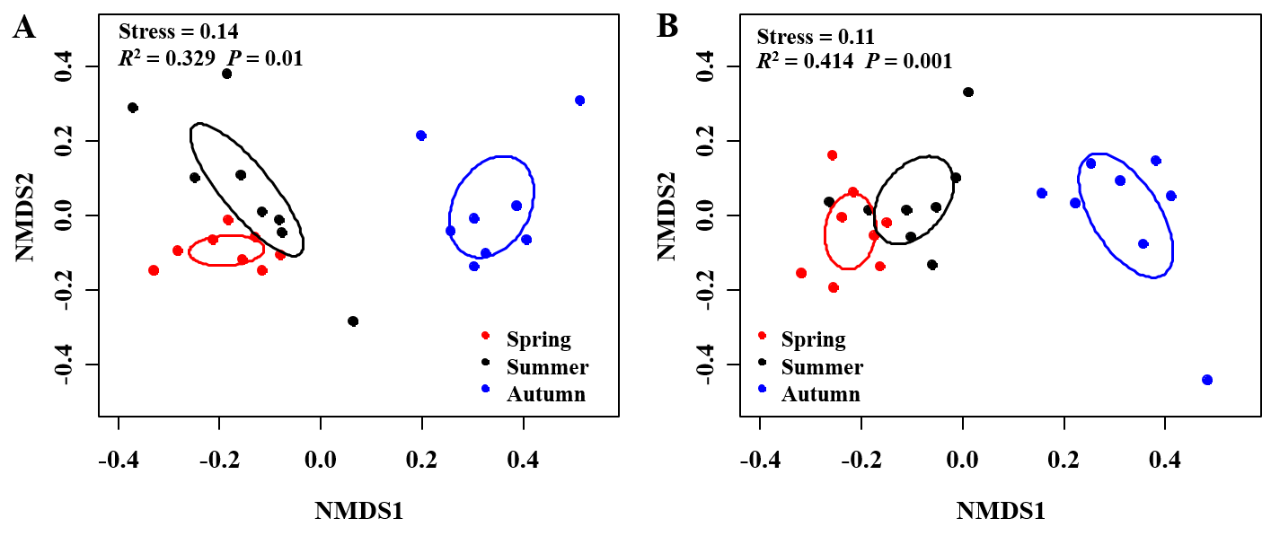


**Supplementary Figure 3.** Nonmetric multidimensional scaling (NMDS) ordinations of saprotrophic (A) and pathogenetic (B) fungal community compositions based on Bray-Curtis distance among seasons. Ellipses delimit 95% confidence intervals around centroids for each season


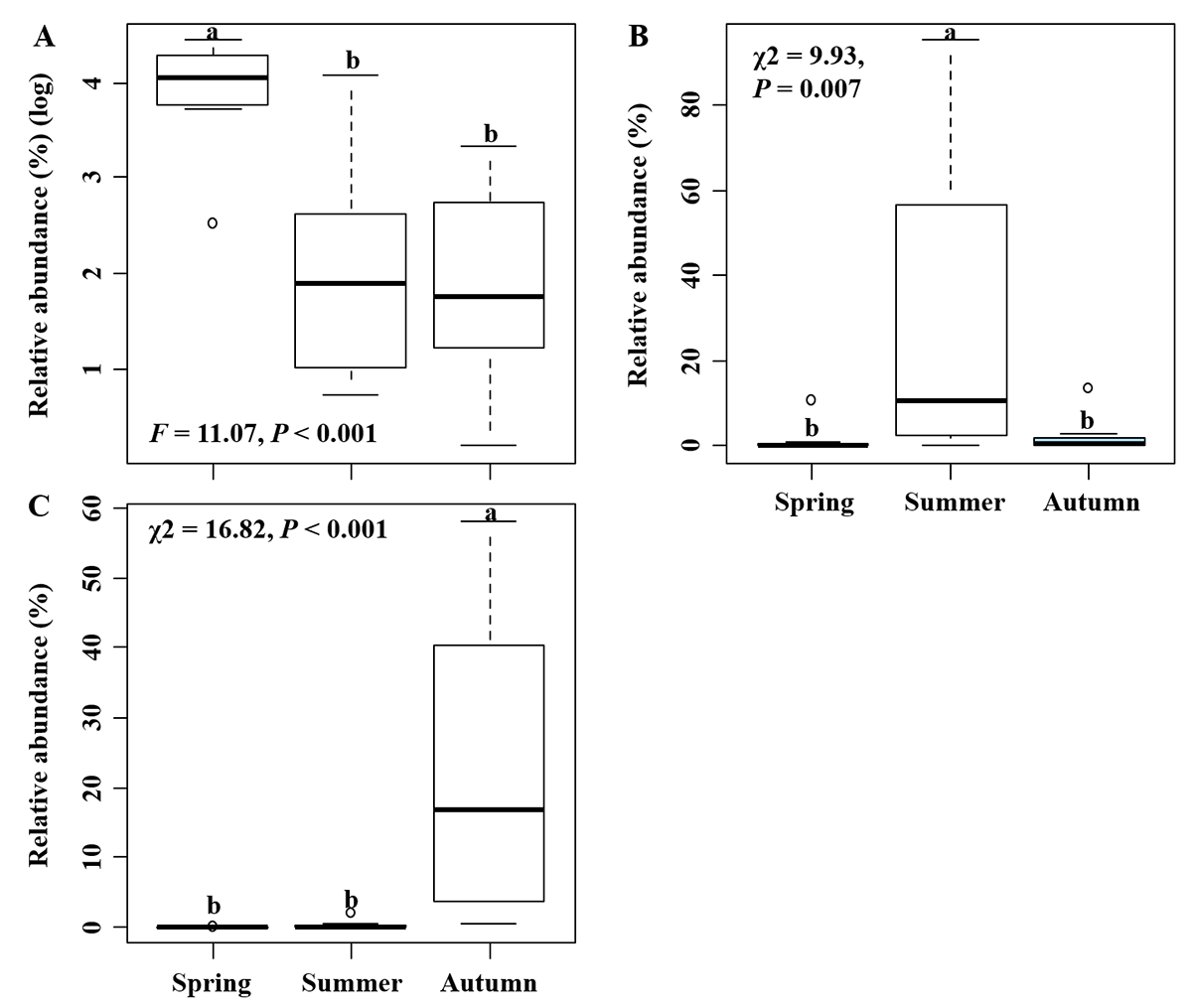


**Supplementary Figure 4.** Boxplots showing the multiple comparison of the relative abundance of abundant fungal classes (> 5% of total sequences). **(A)** Sordariomycetes, **(B)** Agaricomycetes and **(C)** Leotiomycetes. Bars without shared letters indicate significant differences according to Tukey’s HSD or Dunn’s tests with Bonferroni adjustment at *P* < 0.05.


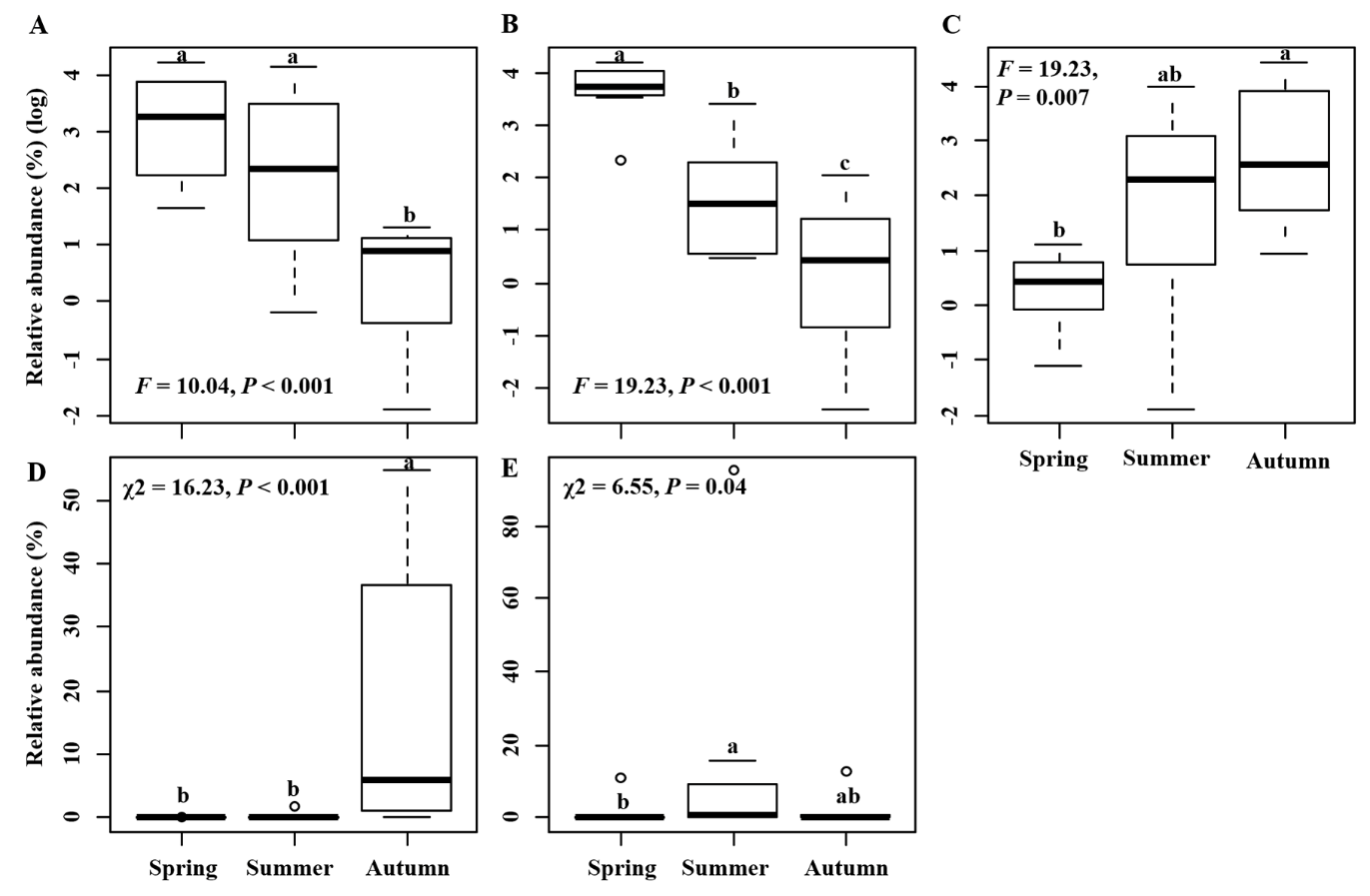


**Supplementary Figure 5.** Boxplots showing the multiple comparison of the relative abundance of abundant fungal orders (> 5% of total sequences). **(A)** Eurotiales, **(B)** Hypocreales, (C) Chaetothyriales, **(D)** Helotiales and **(E)** Agaricales. Bars without shared letters indicate significant differences according to Tukey’s HSD or Dunn’s tests with Bonferroni adjustment at *P* < 0.05.


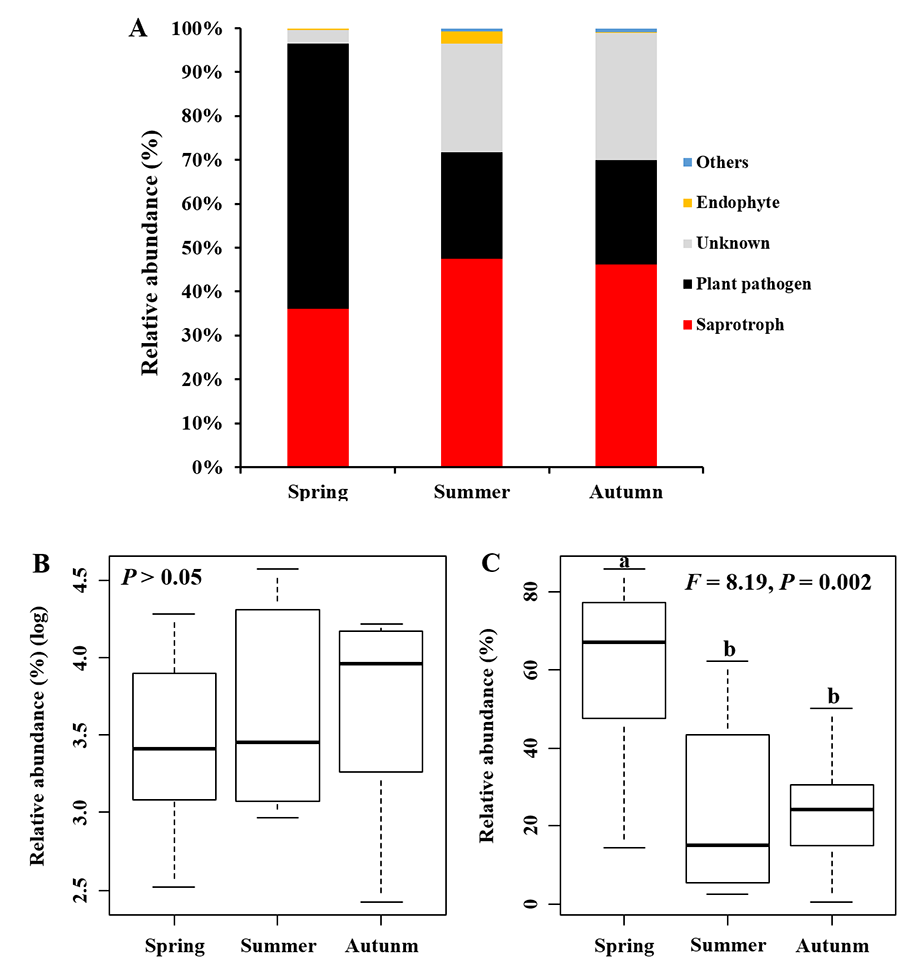


**Supplementary Figure 6.** Dominant fungal guilds (>1% of total reads) **(A)**. Multiple comparison of the relative abundance of saprotrophic **(B)** and pathogenic fungi **(C)**. Bars without shared letters indicate significant differences according to Tukey’s HSD with Bonferroni adjustment at *P* < 0.05.


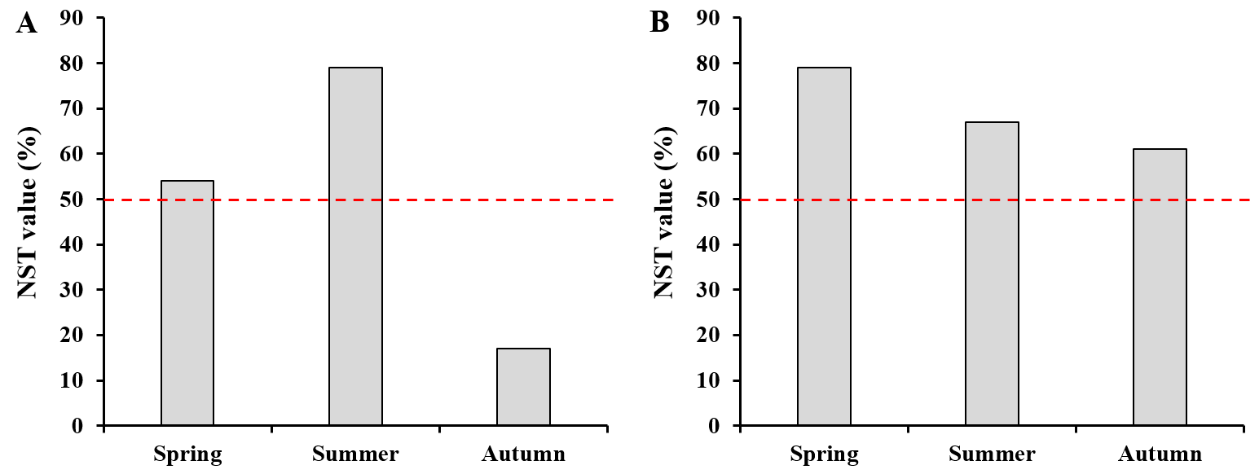


**Supplementary Figure 7.** Normalized stochasticity ratio (NST) showing community assembly pattern of root-associated saprotrophic (A) and pathogenetic (B) fungi of *P. mongolica* across three consecutive seasons.
